# Neuromuscular and Balance Performance Across the Fundamental Preparation Period in Elite Athletes with Lower Limb Deficiencies: A Retrospective Case Series

**DOI:** 10.3390/sports14040144

**Published:** 2026-04-07

**Authors:** Luca Cavaggioni, Athos Trecroci, Raffaele Scurati, Massimiliano Tosin, Linda Casalini, Paolo Castiglioni, Giampiero Merati, Damiano Formenti

**Affiliations:** 1 Department of Theoretical and Applied Sciences, eCampus University, 22060 Novedrate, Italy; 2 Laboratory of Nutrition and Obesity Research, IRCCS Istituto Auxologico Italiano, 20145 Milan, Italy; 3 Department of Biomedical Sciences for Health, Università degli Studi di Milano, 20133 Milan, Italy; athos.trecroci@unimi.it; 4 POLHA-Varese, Associazione Polisportiva per Disabili, 21100 Varese, Italy; max.tosin@gmail.com; 5 Department of Biotechnology and Life Sciences, University of Insubria, 21100 Varese, Italy; linda.casalini@uninsubria.it (L.C.); paolo.castiglioni@uninsubria.it (P.C.); giampiero.merati@uninsubria.it (G.M.); damiano.formenti@uninsubria.it (D.F.); 6 Italian Paralympic Committee, 00191 Rome, Italy; 7 IRCCS Fondazione Don Carlo Gnocchi, 20148 Milan, Italy

**Keywords:** Paralympic, athletes with disabilities, neuromuscular performance, balance, resistance training, retrospective case series

## Abstract

Optimizing neuromuscular strength and balance is essential for performance and injury prevention in elite Paralympic sport. However, limited evidence describes how these parameters change over time during specific phases of the training season in athletes with lower limb deficiencies. This retrospective case series aimed to describe longitudinal changes in neuromuscular and balance performance during the fundamental preparation period in elite athletes using prosthetic devices. Routinely collected performance data from five international-level Paralympic athletes (Para-swimming and Para-athletics) were retrospectively analyzed across two preparatory observation windows conducted in consecutive competitive seasons. Neuromuscular performance was assessed using countermovement jump variables, while static balance was evaluated through Inertial Measurement Unit-derived sway metrics. Within-athlete changes were examined using descriptive and exploratory analyses. At the group level, changes were observed in selected neuromuscular and balance outcomes over time, including jump height and path length. Individual analyses revealed substantial inter-athlete variability in the magnitude and direction of changes across all outcomes. Overall, the findings indicate that neuromuscular and postural performance may fluctuate meaningfully during preparatory phases in elite athletes with lower limb deficiencies. This study provides exploratory insights derived from real-world training settings and highlights the value of longitudinal monitoring to support individualized performance management in Paralympic sport.

## 1. Introduction

Optimizing physical performance while preserving overall well-being represents a central objective in high-level athletic preparation [1]. Although evidence indicates that Olympic and Paralympic athletes often adopt comparable training frameworks and performance models [2], athletes with disabilities require additional considerations to ensure inclusive, individualized, and sustainable approaches that adequately address both physical and psychological demands [3,4].

Among Paralympic athletes with lower limb impairments using prosthetic devices, deficits in muscular strength and balance are consistently reported as major functional challenges [5,6,7]. Strength and power outputs are frequently reduced, particularly in the affected limb, leading to altered force production and inter-limb asymmetries [8,9]. In athletes with transtibial amputations, these impairments are most pronounced in the knee flexor and extensor muscle groups of the residual limb [8], whereas individuals with transfemoral amputations commonly exhibit greater weakness at the hip level [6,8]. Such neuromuscular limitations may influence both athletic performance and the interaction between the athlete and the prosthetic device. Within this context, the systematic monitoring of neuromuscular and postural variables throughout the training season is crucial for understanding functional adaptations and supporting informed decision-making in elite sport environments.

Balance and postural control are also adversely affected by the loss of sensory and proprioceptive input from the amputated limb [10]. As a result, athletes often adopt compensatory postural strategies, including reduced reliance on the prosthetic limb during static stance and dynamic tasks such as walking or running [11,12,13]. Previous research has documented an anterior displacement of the center of pressure toward the amputated side, accompanied by increased anteroposterior sway, reflecting altered postural control mechanisms in this population [14]. These neuromuscular and balance impairments may contribute to the development of asymmetrical patterns during sport-specific activities, particularly in disciplines such as running and swimming [15,16]. Both activities require high levels of whole-body strength, power, and postural stability to achieve optimal competitive performance [17,18,19]. Moreover, neuromuscular and postural capacities are not static and may fluctuate across the training season, especially during the fundamental preparation period, when training loads and stimuli are progressively increased [20]. This variability highlights the importance of longitudinal performance monitoring to capture meaningful changes over time and to support individualized training management in elite Paralympic athletes.

While strength and balance are critical for injury prevention and performance in Paralympic sport, little is known about how these parameters fluctuate throughout specific phases of the training season in athletes with lower limb deficiencies. In particular, few studies have examined longitudinal changes in neuromuscular and balance performance using data collected under real-world training conditions [21,22]. For example, Edmonds et al. [21] monitored daily heart rate variability in Paralympic swimmers across a 17-week training period, whereas Kasińska et al. [22] investigated preseason balance assessments in amputee football players to explore injury risk. Although, retrospective analyses of routinely collected measurements may be affected by potential biases related to the use of previously collected data, this approach may offer valuable insights into functional adaptations occurring during preparation phases, while the minimizing disruption to athletes’ training processes [23].

The present study intends to fill this gap by providing a retrospective case series examining neuromuscular strength and balance performance in elite Paralympic athletes with lower limb deficiencies who use prosthetic devices. By analyzing performance data collected at four predefined time points during preparatory phases of two consecutive competitive seasons, this study aims to describe within-athlete changes in neuromuscular and postural control over time, thereby contributing to the limited body of literature on performance monitoring and management in elite athletes with physical disabilities.

## 2. Materials and Methods

### 2.1. Participants

Given the retrospective nature of this case series, no priori sample size calculation was performed. Despite the limited sample size, this type of case series methodology is commonly applied in studies involving elite or unique athletes, where the value lies in detailed case descriptions and the generation of practical insights. A post hoc exploratory analysis based on previous literature examining countermovement jump performance in trained individuals was conducted solely to situate the sample size within the existing body of evidence, and not to infer statistical power [24].

Initially, 11 athletes were screened. Eligibility criteria were applied retrospectively to define the study population for descriptive analysis. Inclusion criteria required athletes to be classified with a limb deficiency, to have participated in at least one international-level competition during the four-year Paralympic cycle, and to have a minimum of three years of resistance training experience. Cases were excluded with the presence of an acute upper-limb injury on the day of testing, the presence of motor, sensory, or intellectual disabilities other than lower limb deficiency, or any clinical condition affecting regular training practice. According to the eligibility criteria, four athletes were excluded, and two withdrew during data collection for external reasons.

The final sample consisted of five international-level elite athletes with lower limb deficiencies (age: 25.8 ± 3.1 years; height: 1.77 ± 0.06 m; body mass: 71.3 ± 4.4 kg), competing in Para-swimming (*n* = 3) and Para-athletics (*n* = 2). Athlete-specific characteristics were as follows: Athlete 1 (A1), male swimmer with congenital left femoral hypoplasia (classification code S9–SM9); Athlete 2 (A2), male swimmer with congenital right femoral hypoplasia and coxa vara (classification code S9–SM9); Athlete 3 (A3), male swimmer with right transfemoral amputation (classification code S8); Athlete 4 (A4), male sprinter with left below-knee amputation (classification code T64); and Athlete 5 (A5), male sprinter with right below-knee amputation (classification code T64).

Written informed consent was obtained from all participants. All procedures were conducted in accordance with the Declaration of Helsinki and approved by the Ethics Committee of the University (approval number 0130560).

### 2.2. Study Design and Setting

This study was designed as a retrospective case series based on the a posteriori analysis of routinely collected performance data obtained during athletes’ fundamental preparation period. Data were originally collected as part of the team’s routine performance monitoring procedures. Ethical approval for the retrospective use and analysis of these data was obtained from the institutional ethics committee (approval date: 22 December 2025). Repeated neuromuscular and balance assessments were available for each athlete at predefined time points, allowing within-athlete comparisons over time.

**Figure 1:**
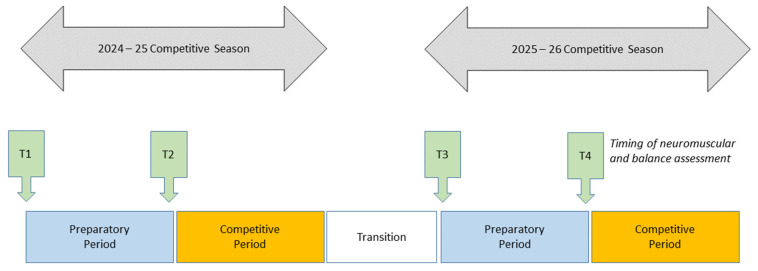
Timeline of neuromuscular and balance performance assessments collected during preparatory phases of two consecutive competitive seasons. T1, T2, T3 and T4 are the time points at which the neuromuscular and balance measures were taken. Assessments were performed for observational purposes only. No experimental manipulation or randomization was applied.

Data were collected across two preparatory observation windows, each lasting eight weeks. Specifically, assessments conducted at T1 and T2 were obtained during the preparatory period of the 2024–2025 competitive season, whereas assessments at T3 and T4 were collected during the preparatory period of the subsequent 2025–2026 season (Figure 1). These time points were not defined as priori for research purposes but as reflected routine performance monitoring practices adopted by the coaching staff within the athletes’ training programs, typically conducted at the beginning and at the end of each preparatory phase to monitor short-term adaptations. Although data collection spanned two competitive seasons, all assessments were conducted within comparable preparatory contexts and were included to describe longitudinal changes in neuromuscular and balance performance over time. No experimental manipulation or randomization was applied.

All testing procedures were conducted in the same indoor gym facility under comparable environmental conditions (temperature: 21–25 °C; relative humidity: 40–50%) and at the same time of day (between 2:00 p.m. and 4:00 p.m.). The prosthesis was not used during the CMJ and balance assessments. Athletes wore a running shoe on the sound limb, and all tests were conducted on a rubber gym surface. Trials were deemed invalid if any of the following criteria were met: (i) excessive forward trunk lean during the braking phase; (ii) absence of a clear transition between the eccentric and concentric phases; or (iii) landing in a position different from the take-off location.

The warm-up protocol consisted of 5 min of cycling on a cycle ergometer at a self-selected moderate intensity, followed by 5 min of dynamic stretching involving both upper and lower limbs, and finally a series of jump variations performed at a progressively increasing, self-selected intensity.

To minimize the effects of residual fatigue, the testing sequence was standardized, with balance assessment performed first, followed by CMJ evaluation.

Athletes were instructed to abstain from alcohol and caffeinated beverages prior to testing, to maintain their habitual meals, and to refrain from exhaustive physical activity, training sessions, or competitions during the 24 h preceding each assessment. This study is reported according to the STROBE statement; the completed checklist is provided within the Supplementary Material (File S1). 

### 2.3. Neuromuscular Performance Measure

Neuromuscular performance was assessed using a countermovement jump with arm swing, which is positively associated with dynamic athletic performance [25]. Although the use of arm swing may increase variability, it was included to allow a natural execution of the movement and to maximize performance output, as commonly adopted in performance testing protocols [26,27]. To reduce variability, standardized cues to jump “as high and as fast as possible” were maintained throughout the testing procedure to minimize its influence on jump outcomes.

A wireless, wearable, Inertial Measurement Unit (IMU) sensor (Gyko system, Microgate, Bolzano, Italy) with previously established validity and reliability for jumping performance was adopted [25,28]. The IMU was positioned at the lumbar spine using a dedicated belt. The Gyko system integrates a triaxial accelerometer, gyroscope, and magnetometer (full-scale range: ±16 g; angular velocity up to 2000 °/s) and records data at a sampling frequency of 1000 Hz. Signals were transmitted via Bluetooth to a personal computer and processed using dedicated software (Gyko RePower Software, version 1.2.2.0). The software automatically calculated peak eccentric work (EW), peak concentric work (CW), peak power (PP), and jump height (JH), with jump height corrected using the appropriate equation [25].

Three countermovement jump trials were performed, with a 120 s rest interval between trials. For each variable (EW, CW, PP, JH), the best trial was selected for subsequent analyses.

### 2.4. Balance Performance Measure

Static balance performance was assessed by measuring body sway using the same wearable inertial sensor (Gyko system, Microgate, Bolzano, Italy), positioned at the T1–T2 thoracic level using a chest strap [28]. The sensor records trunk accelerations and angular velocities, which reflect the displacement of the body center of mass during quiet standing. These signals were processed using the manufacturer’s proprietary algorithms to derive sway-related parameters equivalent to center-of-pressure metrics. This method has demonstrated to be valid at detecting balance performance [28] with moderate-to-good reliability (intraclass correlation coefficient: 0.62–0.70) [28].

Sensor signals were transmitted via Bluetooth to a personal computer, and the Gyko Re-Power Software automatically calculated standard postural stability parameters, including IMU-derived sway metric ellipse area (cm^2^), path length (cm), and path velocity (cm/s). Athletes were instructed to stand barefoot on their sound limb in an upright position with eyes open and arms relaxed at their sides for 30 s. Three trials were performed with 60 s of rest between trials. The best trial for each variable (ellipse area, path length, path velocity) was retained for analysis.

### 2.5. Training Background

During the observed period, athletes followed their usual resistance training programs as prescribed and supervised by a qualified sport scientist with a specific certification in inclusive fitness training (ACSM/NCHPAD Inclusive Fitness Specialist). Training sessions were held three times per week and included exercises to develop muscular strength and power using free weights, cable machines, elastic bands, and medicine balls.

During the preparatory observation window corresponding to T1–T2 (2024–2025 season), resistance training programs featured the concurrent development of maximal strength and power within the same training week, with frequent variation in training stimuli across sessions.

In contrast, during the preparatory observation window corresponding to T3–T4 (2025–2026 season), resistance training programs were structured more sequentially, with distinct phases emphasizing maximal strength followed by phases emphasizing power-oriented exercises.

Exercise selection included squats, upper-body pushing and pulling movements, torso rotation exercises, and Olympic lift derivatives (e.g., clean and press, snatch). Detailed descriptions of the training content and the weekly structure are reported in Table 1 and Table 2, respectively. Training variables were not experimentally manipulated for the purpose of the present study and are reported solely to provide contextual information regarding the athletes’ preparation [3,29].

### 2.6. Statistical Analysis

Given the exploratory nature of this retrospective case series and the small sample size, statistical analyses were primarily descriptive. Data distribution was assessed using the Shapiro–Wilk test. Results are also presented at both the individual and group levels to facilitate the interpretation of longitudinal response patterns. The intra-class correlation coefficient (ICC) and the coefficient of variation (CV) were calculated for reliability purposes for all variables.

Based on the normality test results, exploratory statistical comparisons between percentage changes from T1 to T2 (delta) and from T3 to T4 were performed using paired Student’s t test for all parameters, and using Wilcoxon matched-pairs signed-rank test for peak eccentric work (EW). In addition, the magnitude of the effect was computed with Cohen’s (d) effect size [30], and the non-parametric effect size (ES) *r* was calculated as the ratio between the Z-score statistics and the square root of the sample size [31].

Given the small sample size and the exploratory nature of the study, *p*-values are reported for descriptive purposes only and should not be interpreted as evidence of inferential statistical significance. All analyses were conducted using the Statistical Package for Social Sciences (IBM SPSS Statistics, version 21.0; IBM Corp., Chicago, IL, USA). Statistical significance was set at *p* < 0.05 for exploratory purposes only.

In addition, a magnitude-based approach was used as a complementary framework to aid the practical interpretation of observed changes based on the concept of the smallest worthwhile change (SWC), defined as one-third of the coefficient of the variation in each performance variable [32]. This approach was employed to describe the likelihood and direction of observed changes rather than providing inferential conclusions. Individual calculations were performed using a customized spreadsheet available at www.sportsci.org (accessed on 29 January 2026).

## 3. Results

All five athletes completed the observation period without the need for modifications to their usual training routines, and no adverse events were reported during the data collection timeframe. Adherence to the prescribed training sessions during the observation period was high across participants, ranging from 93.7% to 100%. Both ICC and CV results showed a higher reliability for each IMU-derived variable: peak eccentric work (ICC = 0.99 [0.99–1.0]; CV = 2.9%), peak concentric work (ICC = 0.88 [0.54–0.98]; CV = 4.6%), peak power (ICC = 0.99 [0.99–1.0]; CV = 3.6%), jump height (ICC = 0.92 [0.67–0.99]; CV = 3.0%), ellipse area (ICC = 1.0 [0.99–1.0]; CV = 1.6%), path length (ICC = 0.99 [0.99–1.0]; CV = 2.0%), and path velocity (ICC = 0.98 [0.97–0.99]; CV = 3.7%)

### 3.1. Group Changes in Neuromuscular and Balance Outcomes

At the group level, within-athlete comparisons between the two observation time points revealed a significant difference in JH and path length. For the other IMU derived metrics it is possible to appreciate a trend toward significance (Table 3).

### 3.2. Individual Athlete Responses

**Figure 2:**
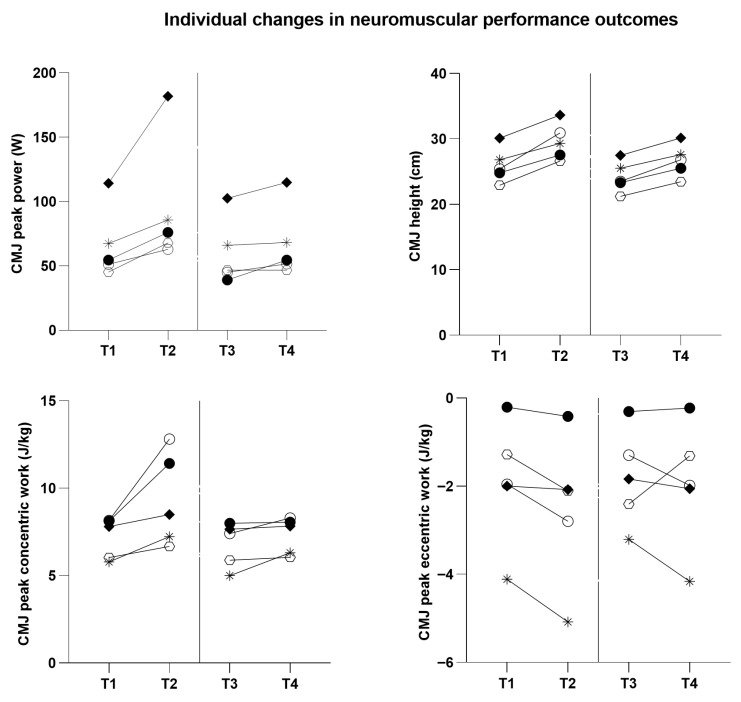
Individual changes in countermovement jump (CMJ) performance outcomes across predefined assessment time points (T1–T4) during the fundamental preparation period. Each symbol represents a single athlete: A1 (●), A2 (*), A3 (○), A4 (◆), and A5 (⬡). The vertical dashed line separates the two preparatory observation windows (T1–T2 and T3–T4) and is provided for temporal reference only.

**Figure 3:**
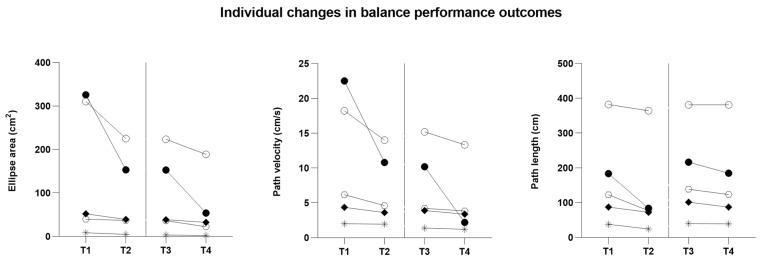
Individual changes in balance performance outcomes (center of pressure, COP) across predefined assessment time points (T1–T4) during the fundamental preparation period. Each symbol represents a single athlete: A1 (●), A2 (*), A3 (○), A4 (◆), and A5 (⬡). The vertical dashed line separates the two preparatory observation windows (T1–T2 and T3–T4) and is provided for temporal reference only; it does not indicate experimental manipulation.

Individual responses for countermovement jump variables and static balance outcomes are illustrated in Table 4 and Figure 2 and Figure 3, highlighting inter-individual variability in the magnitude and direction of change.

As Table 4 and Table S1 report, athlete A1 showed clear changes in countermovement jump variables across both observation windows, with increases in peak power and jump height; balance outcomes indicated reductions in IMU-derived sway metrics, suggesting improved postural stability over time. Athlete A2 demonstrated improvements in countermovement jump performance, particularly in peak power and jump height; balance parameters also changed across time points, indicating modifications in postural control. Athlete A3 showed changes across all countermovement jump variables during the observation period, including eccentric work, concentric work, peak power, and jump height; balance outcomes also demonstrated reductions in IMU-derived sway metrics at the later assessment points, suggesting improved postural stability over time. Athlete A4 demonstrated changes in countermovement jump performance across the two observation windows, particularly in peak power and jump height; static balance parameters also decreased over time, indicating potential improvements in postural control. Athlete A5 exhibited changes across all countermovement jump variables during the observation period; balance parameters also varied between assessment points, with reductions in IMU-derived sway metrics suggesting decreased postural sway and potential improvements in postural stability.

## 4. Discussion

This retrospective case series study aimed to describe changes in neuromuscular and balance performance in elite athletes with lower limb deficiencies across the distinct phases of their fundamental preparation period. By analyzing routinely collected performance data at predefined time points, this study provides exploratory insights into within-athlete adaptations in strength- and balance-related outcomes in high-level Paralympic athletes.

At the group level, within-athlete analyses revealed measurable changes in selected countermovement jump and balance parameters over time. Significant variations were observed in countermovement jump height and path length in balance assessment, while the other IMU derived metrics showed a tendency towards significance. This indicates that both neuromuscular and postural control capacities may fluctuate during short-term preparatory phases. These findings are consistent with the notion that neuromuscular performance is sensitive to changes in training content particularly in elite athletes [33,34].

Beyond group-level observations, individual athlete responses highlighted substantial inter-individual variability in the magnitude and direction of change across neuromuscular and balance outcomes. Such individual variability has been consistently reported in high-performance sport settings and emphasizes the importance of considering individual trajectories rather than relying exclusively on aggregated group data [35,36,37]. Variability in training stimuli may contribute to divergent adaptive responses, with some athletes exhibiting more pronounced changes and others showing modest or mixed responses [32,38].

The observed patterns are broadly consistent with previous research in non-disabled trained individuals, in which greater variability in resistance training stimuli has been associated with enhanced neuromuscular adaptations compared to more uniform training structures [39,40,41]. Nevertheless, given the retrospective design of the present case series, these observations should be interpreted cautiously and cannot be fully attributed to a specific training structure. From a physiological perspective, early changes in strength and power are often attributed predominantly to neural adaptations rather than morphological changes [42]. Increased variability in training demands may promote enhanced neural drive, motor unit recruitment, and coordination, potentially contributing to short-term performance improvements [43]. Although no direct assessments of neural function were performed in the present study, these proposed mechanisms should be regarded as speculative in the context of this descriptive case series.

Existing literature suggests that different training structures—with a comparable training load—may elicit similar adaptations in well-trained athletes [44,45,46]. Although training load was not quantified in the present study, it is plausible that the adaptations occurred in neuromuscular and balance parameters may be mainly attributed to training structure. In this context, training background, competitive level, and prior resistance training experience may attenuate responsiveness, leading to similar neuromuscular outcomes across different preparatory approaches [47,48]. These considerations are particularly relevant in elite Paralympic athletes, whose adaptive capacity may be influenced by both long-term training exposure and impairment-specific constraints.

In Paralympic sport, fundamental training principles largely overlap with those applied in non-disabled athletes, although greater individualization is often required due to impairment-related factors [2,4,49]. Athletes with lower limb deficiencies frequently present reduced force-generating capacity in the affected limb [50], substantial inter-limb asymmetries [51], and impaired balance control due to altered sensory and proprioceptive feedback [14,52]. In the present study, neuromuscular and balance performance was assessed on the sound limb and the observed changes should be considered in relation to each athlete’s functional profile, daily life prosthesis use, and the unilateral nature of the assessment.

From an applied perspective, the present findings suggest that preparatory phases may be associated with meaningful fluctuations in strength, power, and balance performance in elite athletes with lower limb deficiencies [53,54]. The longitudinal monitoring of these variables may help practitioners to identify individual response patterns and optimize the interaction between the athlete and assistive devices, such as prostheses [35].

Nevertheless, caution is warranted when interpreting these results. Due to the study’s retrospective design and small sample size, causal inferences about specific training structures cannot be drawn. The findings should therefore be considered descriptive and exploratory, aimed at generating hypotheses rather than confirming the superiority of any specific training approach [55].

Future research should employ prospective and adequately powered study designs to further investigate neuromuscular and balance adaptations in athletes with disabilities. In particular, randomized controlled trials and long-term longitudinal studies are warranted to clarify the effects of different training approaches across a broader range of impairments and to determine the durability of observed performance changes.

Various limitations should be acknowledged. First, the small sample size (*n* = 5) and the inclusion of athletes with lower limb impairments may limit the generalizability of the findings. However, recruiting elite Paralympic athletes with comparable performance characteristics remains challenging. Second, a retrospective observational approach was adopted instead of a prospective design due to the elite level of the athletes and the structured nature of their training programs. This precluded the manipulation of training variables and therefore the implementation of prospective research. Third, the absence of long-term follow-up precludes conclusions regarding the persistence of the observed adaptations. Moreover, the lack of direct maximal strength and neuromuscular activation measures limits insight into the underlying mechanisms driving the observed performance changes. Finally, balance assessment was performed on the sound limb only, which may limit transferability to sport-specific conditions. Nevertheless, the sound limb may play a predominant functional role in athletes with unilateral lower limb deficiencies.

## 5. Conclusions

This retrospective case series provides exploratory evidence describing changes in neuromuscular and balance performance in elite athletes with lower limb deficiencies during the fundamental preparation period. The primary finding indicates that measurable variations in neuromuscular and balance performance can be observed over short-term preparatory phases in this specific Paralympic population.

At the individual level, the analysis highlighted considerable inter-athlete variability in neuromuscular and postural responses over time. Several athletes demonstrated pronounced changes in countermovement jump performance and static balance parameters, underscoring the importance of individualized monitoring when evaluating training-related adaptations in athletes with limb deficiencies.

The present findings can contribute to the growing body of literature on performance monitoring in Paralympic sport by offering detailed single-athlete observations in a real-world training context. From an applied perspective, the longitudinal assessment of neuromuscular and balance variables may support practitioners in optimizing training prescription and in refining the interaction between the athlete and assistive equipment, such as prosthetic devices, during preparatory phases.

## Figures and Tables

**Table 1:** Overview of resistance training content during the preparatory phase T1–T2.

Period	Exercise	S	R
	Barbell bench press	6	3
Weeks 1–4; Tuesday (upper-body maximum strength, 80–90% 1RM)	Cable chest press	2	5
	Lat pull-down	6	3
	Row with dumbbells	2	5
	Shoulder lateral raise	3	6
	Triceps cable push down	4	4
	Biceps curl with dumbbells	4	4
	Back squat	6	3
Weeks 1–4; Thursday (lower-body maximum strength, 80–90% 1RM)	Leg press	2	5
	Leg extension	3	6
	Seated leg curl	3	6
	Leg press calf raises	2	8
	Clean and jerk with dumbbell	4	4
	Banded bench press	4	4
Weeks 1–4; Saturday (upper-body explosive strength/power, 40–60% 1RM)	Supine med ball throw	4	6
	Banded pull-down	4	4
	Med ball slam	4	6
	Banded back squat	4	4
	Concentric box jump	2	5
	Snatch with dumbbell	3	3
	Barbell bench press	6	3
Weeks 5–8; Tuesday (upper-body maximum strength, 80–90% 1RM)	Cable chest press	2	5
	Lat pull-down	6	3
	Row with dumbbells	2	5
	Shoulder lateral raise	3	6
	Triceps cable push-down	4	4
	Biceps curl with dumbbells	4	4
	Banded back squat	6	3
Weeks 5–8; Thursday (lower-body explosive strength/power, 40–60% 1RM)	Leg press	2	5
	Banded leg extensions	3	6
	Banded seated leg curl	3	6
	Banded vertical jumps	2	8
	Clean and jerk with dumbbell	4	4
	Banded bench press	4	4
Weeks 5–8; Saturday (upper-body explosive strength/power, 40–60% 1RM)	Supine med ball throw	4	6
	Banded pull-down	4	4
	Med ball slam	4	6
	Banded back squat	4	4
	Concentric box jump	2	5
	Snatch with dumbbell	3	3

Note = 1RM = one repetition maximum; S = sets; Rep = repetitions.

**Table 2:** Overview of resistance training content during preparatory phase T3–T4.

Period	Exercise	S	R
	Barbell bench press	5	5
Weeks 1–4; Tuesday	Lat pull-down	5	5
(maximum strength, 80–90% 1RM)	Triceps cable push down	3	5
	Bicep curl with barbell	3	5
	Shoulder lateral raise	3	5
	Back squat	5	5
	Cable chest press	6	3
Weeks 1–4; Thursday (maximum strength, 80–90% 1RM)	Row with dumbbells	6	3
	Barbell triceps French press	3	5
	Biceps curl with dumbbells	3	5
	Leg press	6	3
	Leg press calf raises	3	5
	Dumbbell bench press	5	5
Weeks 1–4; Saturday (maximum strength, 80–90% 1RM)	Lat pulley	5	5
	Triceps cable push down	5	5
	Biceps curl with cable	3	5
	Leg extension	3	5
	Seated leg curl	3	5
	Banded bench press	4	4
Weeks 5–8; Tuesday (explosive strength/power, 40–60% 1RM)	Supine med ball throw	4	6
	Banded pull-down	4	4
	Med ball slam with two hands	4	6
	Banded back squat	4	4
	Concentric box jump	2	5
	Clap push ups	4	4
Weeks 5–8; Thursday (explosive strength/power, 40–60% 1RM)	Forward med ball throw	4	6
	Cable pull-down	4	4
	Med ball slam with rotation	4	6
	Leg press	4	4
	Banded vertical jumps	2	5
	Banded bench press	4	4
Weeks 5–8; Saturday (explosive strength/power, 40–60% 1RM)	Supine med ball throw	4	6
	Banded pull-down	4	4
	Med ball slam with one hand	4	6
	Banded back squat	4	4
	Concentric box jump	2	5

Note = 1RM = one repetition maximum; S = sets; Rep = repetitions.

**Table 3:** Percent changes in neuromuscular and balance performance outcomes across preparatory observation windows as mean (sd) with *p*-values after paired T-test and Cohen’s d effect size if the distributions passed the Shapiro–Wilks normality test, otherwise as median [IQR] with *p*-values after paired Wilcoxon sign rank test and |r| effect size.

Performance	Change T1–T2 (%)	Change T3–T4 (%)	*p*-Value (Exploratory)	Effect Size
Neuromuscular				
CMJ peak power (PP)	40% (15%)	14% (16%)	0.062	Large, 1.15
CMJ height (JH)	14% (5%)	10% (2%)	0.047	Large, 1.27
CMJ peak concentric work (CW)	28% (20%)	9% (11%)	0.11	Large, 0.92
CMJ peak eccentric work (EW)	43% [41%]	12% [55%]	0.81	Small, 0.11
Balance				
Ellipse area	−32% (18%)	−34% (20%)	0.82	Very small, 0.11
Path velocity	−24% (18%)	−25% (30%)	0.92	Very small, 0.04
Path length	−30% (19%)	−9% (7%)	0.045	Large, 1.29

Note that *p*-values and effect sizes are reported for exploratory purposes to describe changes over time: no inferential comparisons between preparatory periods were intended. CMJ: countermovement jump.

**Table 4:** Neuromuscular and balance performance individual changes across predefined assessment time points (T1–T4).

	Countermovement Jump								IMU-Derived Sway Metric					
	Eccentric Work		Concentric Work		Peak Power		Height		Ellipse Area		Path Velocity		Path Length	
Athlete	T_2_–T_1_	T_4_–T_3_	T_2_–T_1_	T_4_–T_3_	T_2_–T_1_	T_4_–T_3_	T_2_–T_1_	T_4_–T_3_	T_2_–T_1_	T_4_–T_3_	T_2_–T_1_	T_4_–T_3_	T_2_–T_1_	T_4_–T_3_
A1	↓ ↔	↑ ↔	↑ *	↑ ?	↑ *	↑ ↔	↑ ↔	↑ ↔	↓ *	↓ *	↓ *	↓ *	↓ *	↓ ↔
A2	↓ ↔	↓ ↔	↑ ↔	↑ ↔	↑ *	↑ ?	↑ ↔	↑ ↔	↓ ↔	↓ ?	↓ ?	↓ ?	↓ ↔	↑ ?
A3	↓ *	↓ ↔	↑ *	↑ ↔	↑ ↔	↑ ↔	↑ *	↑ ↔	↓ *	↓ ↔	↓ *	↓ ?	↓ ↔	↓ ?
A4	↓ ?	↓ ↔	↑ ↔	↑ ?	↑ *	↑ ↔	↑ ↔	↑ ↔	↓ ↔	↓ ?	↓ ↔	↓ ↔	↓ ↔	↓ ↔
A5	↓ *	↑ *	↑ ↔	↑ ?	↑ *	↑ ?	↑ ↔	↑ ↔	↓ ?	↓ ↔	↓ ↔	↓ ?	↓ *	↓ ↔

Note = Symbols used in this table follow the notation provided in the spreadsheet available at www.sportsci.org (accessed on: 29 January 2026); symbol ↔ represents a *clear* change; symbol ? represents an *unclear* change; symbol * represents a *very likely* change; symbol ↑ represents a *substantial increase*; symbol ↓ represents a *substantial decrease*.

## Data Availability

The data are not publicly available due to privacy reasons. Data may be available upon reasonable request to the Corresponding Author.

## Supplementary Materials

The following supporting information can be downloaded at: https://www.mdpi.com/article/10.3390/sports14040144/s1, “File S1: STROBE Statement—checklist of items that should be included in reports of observational studies”. “Table S1: Chance as a percentage to obtain a *substantial decrease/trivial/substantial* increase in neuromuscular or balance performance” can be downloaded from the Zenodo repository at https://doi.org/10.5281/zenodo.18803571 (accessed on 25 March 2026).
